# The Marine Compound Isaridin E Ameliorates Lipopolysaccharide-Induced Vascular Endothelial Inflammation via the Downregulation of the TLR4/NF-κB Signaling Pathway

**DOI:** 10.3390/md23040145

**Published:** 2025-03-28

**Authors:** Jing Liu, Xin Zeng, Yu-Quan Lin, Yu-Sheng Peng, Lan Liu, Sen-Hua Chen, Yan-Hua Du

**Affiliations:** 1 Department of Pharmacology, Zhongshan School of Medicine, Sun Yat-sen University, Guangzhou 510080, China; 2 Department of Pharmacy, The Second Clinical Medical College, Southern Medical University, Guangzhou 510280, China; 3 School of Marine Sciences, Sun Yat-sen University, Guangzhou 510275, China; 4 Southern Marine Sciences and Engineering Guangdong Laboratory (Zhuhai), Zhuhai 519000, China

**Keywords:** isaridin E, marine natural products, endothelial inflammation, TLR4, NF-κB

## Abstract

Isaridin E, a cyclodepsipeptide derived from the marine fungus *Beauveria felina* (SYSU-MS7908), has been demonstrated to possess multiple biological properties. In this study, we employed both lipopolysaccharide (LPS)-stimulated human umbilical vein endothelial cells (HUVECs) and a LPS-induced murine endotoxemia model to investigate its anti-inflammatory effects. Our results revealed that isaridin E suppressed the expression of pro-inflammatory cytokines and adhesion molecules in a concentration dependent manner, while also reducing monocyte adhesion to endothelial cells. Furthermore, this compound attenuated vascular hyperpermeability and inflammatory cell infiltration in the lungs, as well as preserving the integrity of the aortic and pulmonary tissues. At the molecular level, isaridin E was found to downregulate TLR4 expression, increase IκBα levels, and inhibit the LPS-induced phosphorylation and nuclear translocation of NF-κB p65. In conclusion, our findings indicate that isaridin E exerts robust anti-inflammatory effects in LPS-induced endotoxemia through the suppression of the TLR4/NF-κB signaling axis, positioning it as a promising therapeutic candidate for vascular inflammatory disorders.

## 1. Introduction

Vascular inflammation serves as a common early pathological event in the development of atherosclerosis, diabetes-associated vascular complications, and sepsis, acting as a precursor to accelerated atherogenesis and multi-organ dysfunction [1]. The vascular endothelium, a critical interface between circulating blood and peripheral tissues, plays a crucial role in vascular inflammation. Endothelial activation, characterized by the upregulation of cell adhesion molecules (such as VCAM-1 and ICAM-1) and pro-inflammatory cytokines and chemokines (such as IL-6, TNF-α, and MCP-1), represents a central feature of vascular inflammatory pathologies [2,3]. The dysregulated activation of endothelial cells leads to enhanced leukocyte adhesion and impaired endothelial barrier integrity. These perturbations disrupt endothelial homeostasis, accelerate atherosclerotic plaque formation, and contribute to end-organ damage in diverse inflammatory disorders [4]. Consequently, therapeutic strategies targeting endothelial inflammatory resolution emerge as essential paradigms for mitigating vascular dysfunction and tissue injury in inflammation-driven disease states.

Over the past decade, marine ecosystems have emerged as a prolific source of novel bioactive compounds, with natural products from marine organisms yielding a plethora of therapeutically promising agents. Isaridin E, a cyclodepsipeptide isolated from marine-derived fungus *Beauveria felina* (SYSU-MS7908) from the South China Sea, exhibits diverse biological activities. A prior study has established its insecticidal efficacy [5]. In addition, it has been demonstrated that isaridin E remarkably attenuates neutrophil activity via the suppression of formyl-methionyl-leucyl-phenylalanine (FMLP)-induced superoxide anion release [6]. Furthermore, our previous findings revealed that isaridin E exerts excellent antiplatelet and antithrombotic effects, suggesting its multi-targeted therapeutic potential [7]. Endothelial activation is a key initiating factor for platelet adhesion and thrombus formation. More recently, in a murine model of sepsis induced by caecal ligation and puncture (CLP), a study from our group demonstrated the protective effects of isaridin E against sepsis through the suppression of von Willebrand factor (vWF)-mediated platelet–endothelial interactions [8]. While pretreatment with isaridin E substantially attenuated sepsis-associated pulmonary vascular hyperpermeability in this paradigm, its direct modulatory effects on vascular endothelial inflammation and the precise molecular mechanisms underlying these actions remain to be elucidated.

In this study, we aimed to investigate the anti-inflammatory efficacy of isaridin E in both in vitro and in vivo models of lipopolysaccharide (LPS)-induced vascular inflammation, with a focus on its impact on pro-inflammatory cytokine expression, endothelial barrier function, and underlying molecular mechanisms.

## 2. Results

### 2.1. Isaridin E Inhibited the Adhesion of Monocytes to Endothelial Cells

**Figure 1:**
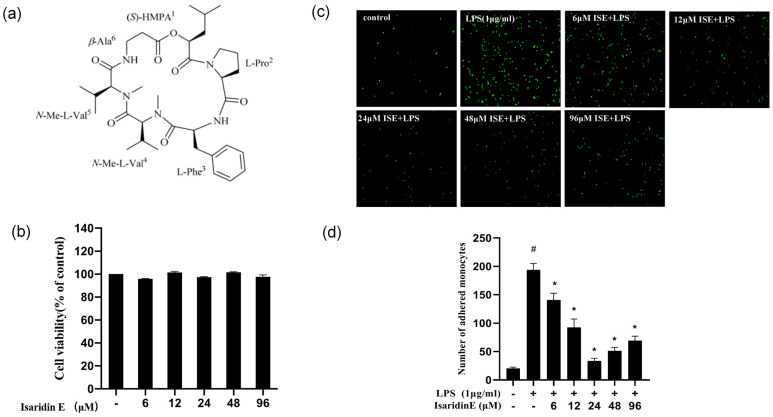
Isaridin E inhibited LPS-induced monocyte adhesion to HUVECs. (**a**) Chemical structure of isaridin E. HMPA (2-hydroxy-4-methylpentanoic acid). (**b**) HUVEC viability was assessed after treatment with isaridin E using the CCK8 assay. Isaridin E (6–96 μm) did not affect the survival rate of HUVECs following 24 h incubation. Results are presented as mean ± SEM (*n* = 6). (**c**) HUVECs were pretreated with isaridin E (6–96 μm) for 2 h before exposure to LPS (1 μg/mL) for 18 h. Representative photomicrographs for adhered THP-1 cells on HUVECs are shown. (**d**) The adhered THP-1 cells with green fluorescence were quantified by counting in five fields randomly chosen from each well. Values are expressed as the mean adhered cell number per optical field. Results are presented as mean ± SEM. (*n* = 6). # *p* < 0.05 vs. LPS (−)/isaridin E (−) group; * *p* < 0.05 vs. LPS (+)/isaridin E (−) group.

The chemical structure of isaridin E is shown in Figure 1a and can also be seen in our previously published paper [7]. Initially, we assessed the impact of isaridin E on the viability of endothelial cells using the CCK-8 assay. The results indicated that isaridin E, evaluated at concentrations ranging from 6 to 96 μm, exhibited no significant cytotoxicity on HUVEC viability following 24 h incubation (Figure 1b). The stimulation of HUVECs with LPS resulted in a significant augmentation of THP-1 monocyte adhesion to the endothelial monolayer, a hallmark of inflammatory activation. However, pretreatment with isaridin E markedly reduced the number of monocytes that adhere to HUVECs in a dose-dependent manner, with the maximal inhibition being observed at a concentration of 24 μm (Figure 1c,d). Notably, isaridin E at 24 μm significantly decreased the LPS-induced monocyte adhesion to 15.7 ± 2.6% of the LPS group.

### 2.2. Isaridin E Decreased LPS-Induced Pro-Inflammatory Cytokine Expression in Endothelial Cells

**Figure 2:**
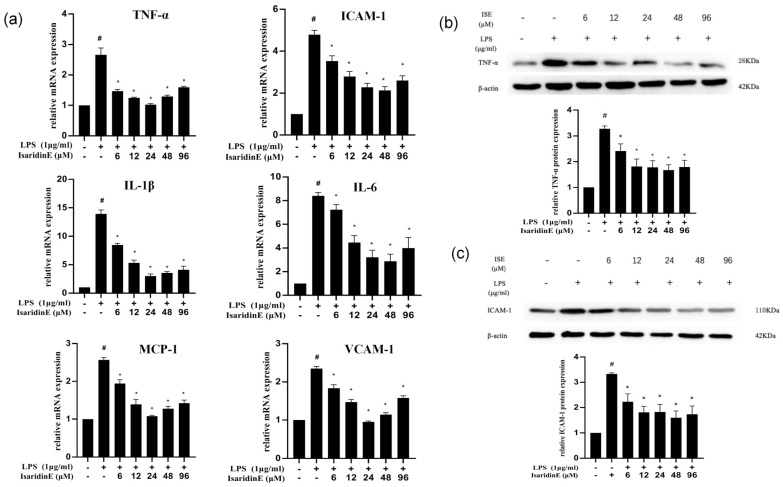
Isaridin E decreased LPS-induced inflammatory response in endothelial cells. (**a**) Isaridin E suppressed the expression of pro-inflammatory cytokines and adhesion molecules. HUVECs were pretreated with isaridin E (6–96 μm) for 2 h before exposure to LPS (1 μg/mL) for 18 h; then, the mRNA levels of TNF-α, ICAM-1, IL-1β, VCAM-1, MCP-1, and IL-6 were determined using real-time PCR. Results are presented as mean ± SEM (*n* = 6). # *p* < 0.05 vs. LPS (−)/isaridin E (−) group; * *p* < 0.05 vs. LPS (+)/isaridin E (−) group. (**b**,**c**) Effects of isaridin E on LPS-stimulated protein expression of TNF-α and ICAM-1. LPS (1 μg/mL)-mediated production of TNF-α (**b**) and ICAM-1 (**c**) in HUVECs was analyzed using Western blot after the treatment of cells with the indicated concentrations of isaridin E for 2 h. Results are presented as mean ± SEM (*n* = 6). # *p* < 0.05 vs. LPS (−)/isaridin E (−) group; * *p* < 0.05 vs. LPS (+)/isaridin E (−) group.

To investigate the impact of isaridin E on LPS-induced endothelial inflammation, we quantified the mRNA expression of key pro-inflammatory mediators, including TNF-α, ICAM-1, IL-1β, VCAM-1, IL-6, and MCP-1, in endothelial cells. As illustrated in Figure 2a, LPS exposure significantly upregulated the mRNA levels of all tested cytokines. However, isaridin E treatment dose-dependently attenuated this inflammatory response, with the maximal inhibitory effects being observed at concentrations of 24–48 μm. To validate these findings at the protein level, we performed immunoblotting for TNF-α and ICAM-1, which confirmed that isaridin E significantly suppressed the LPS-induced expression of these adhesion molecules and cytokines (Figure 2b,c).

### 2.3. Isaridin E Ameliorated LPS-Induced Inflammation in Mouse Aortas

**Figure 3:**
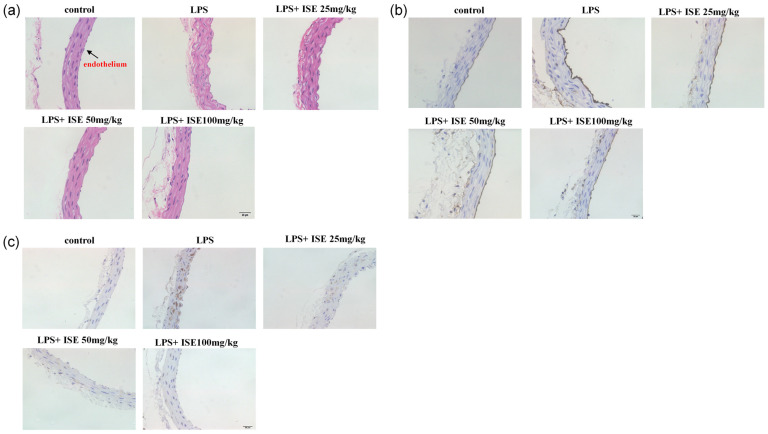
Isaridin E ameliorated LPS-induced inflammation in mouse aortas. (**a**) Representative images of aortic tissue sections stained with hematoxylin–eosin are displayed (400×). (**b**,**c**) Isaridin E attenuated LPS-induced ICAM-1 (**b**) and TNF-α (**c**) expression in the aorta, as was detected using immunohistochemistry. The yellowish-brown deposits referred to the distribution of ICAM-1 and TNF-α positive signals (400×). *n* = 5.

Different doses of isaridin E (25 mg/kg, 50 mg/kg, and 100 mg/kg) were orally administered to mice for three consecutive days, followed by the intraperitoneal injection of LPS to induce inflammation. After a 12 h period, mice were sacrificed and vascular tissues were collected for histological staining. Hematoxylin and Eosin (HE) staining revealed distinct structural differences in aortic morphology across the treatment groups. The control group exhibited normal vascular architecture, characterized by an intact, smooth endothelial monolayer lining the intima and well-ordered smooth muscle cells (SMCs) in the media, with clear demarcation from the adventitia. In contrast, LPS-treated aortas showed marked endothelial damage, characterized by surface protrusions and discontinuities, accompanied by SMC disarray and blurred adventitia–media demarcation. Notably, isaridin E intervention dose-dependently attenuated these pathological changes. In particular, the 100 mg/kg isaridin E group demonstrated the near-complete preservation of endothelial integrity, SMC alignment, and adventitia–media separation, closely resembling control vessels (Figure 3a).

The immunohistochemical staining of aortic cross-sections demonstrated a robust increase in ICAM-1 and TNF-α expression in LPS-treated tissues compared to controls, with immunoreactive deposits appearing as dense, brown–yellow granular aggregates primarily localized to endothelial cells and subendothelial regions. Notably, pretreatment with isaridin E induced a marked attenuation of this inflammatory phenotype relative to LPS-treated samples (Figure 3b,c). These data provide histological evidence that isaridin E effectively counteracts LPS-driven vascular inflammation by downregulating key inflammatory mediators.

### 2.4. Isaridin E Alleviated LPS-Induced Inflammatory Response in Lung Tissues

**Figure 4:**
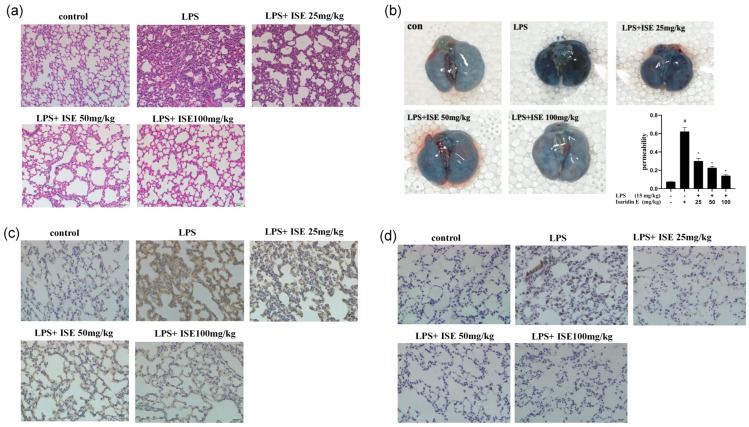
Isaridin E alleviated the LPS-induced inflammatory response in lung tissues. (**a**) The changes in histological structure of mouse lung tissues in different groups (200×). The frozen sections of lung tissues were stained with hematoxylin and eosin for histopathological examination (*n* = 5). (**b**) Effect of isaridin E on LPS-induced Evans Blue accumulation in lung tissues. Evans Blue absorbance was analyzed using spectrophotometry (620 nm). Values are reported as mean ± SEM (*n* = 5). # *p* < 0.05 vs. LPS (−)/isaridin E (−) group. * *p* < 0.05 vs. LPS (+)/isaridin E (−) group. (**c**,**d**) Isaridin E attenuated LPS-induced ICAM-1 and TNF-α expression in the lungs (200×). The distribution of ICAM-1 (**c**) and TNF-α (**d**) expression in lung tissues from distinct experimental groups was detected using immunohistochemistry. Yellowish-brown reaction products referred to ICAM-1 and TNF-α positive signals (*n* = 5).

Enhanced vascular permeability, particularly in pulmonary vasculatures, facilitates the transmigration of erythrocytes and inflammatory cells into the lung interstitium through disrupted endothelial junctions. HE staining demonstrated profound lung parenchymal damage in LPS-challenged animals, characterized by marked alveolar septal thickening, interstitial hemorrhage, and the prominent infiltration of inflammatory cells. In contrast, isaridin E administration conferred dose-dependent protection, as evidenced by the preserved alveolar architecture, mild septal widening, and minimal inflammatory cell infiltration. These histopathological improvements demonstrate the ability of isaridin E to attenuate endothelial barrier dysfunction and suppress LPS-induced pulmonary inflammation (Figure 4a).

In order to further validate the protective effect of isaridin E on vascular barrier integrity during LPS-induced inflammation, the Evans Blue dye extravasation assay was conducted. Quantitative analysis revealed a 6-fold increase in pulmonary Evans Blue accumulation in LPS-treated mice compared to controls, reflecting severe pulmonary microvascular leakage. Notably, isaridin E administration resulted in a dose-dependent reduction in dye leakage, with the highest dose (100 mg/kg) restoring Evans Blue levels to near-baseline values (Figure 4b). These data establish isaridin E as a potent inhibitor of LPS-induced albumin extravasation across the pulmonary endothelium during inflammatory processes. Concurrently, immunohistochemical staining analysis demonstrated that isaridin E significantly attenuated LPS-stimulated ICAM-1 and TNF-α expression in lung tissues (Figure 4c,d). Collectively, these results provide compelling evidence that isaridin E effectively mitigates LPS-driven pulmonary inflammation.

### 2.5. Isaridin E Inhibited LPS-Induced Inflammation in Endothelial Cells Through the TLR4/NF-κB Pathway

**Figure 5:**
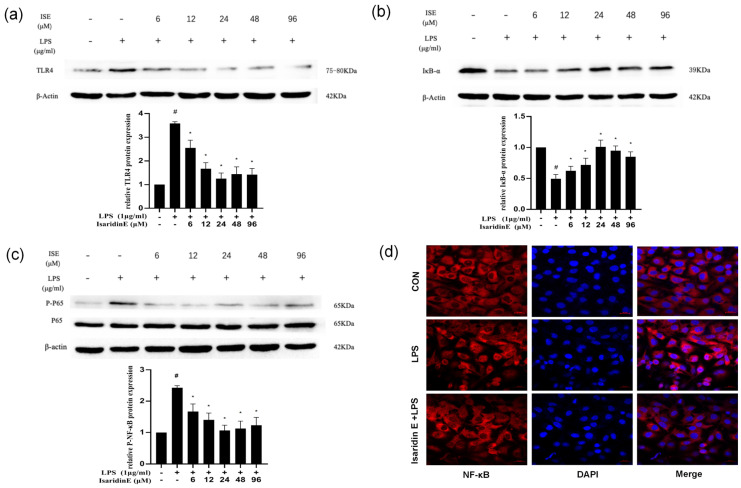
Effects of isaridin E on LPS-induced TLR4/NF-κB signal activation in HUVECs. HUVECs were pretreated with isaridin E (6–96 μm) for 2 h before exposure to LPS (1 μg/mL) for 18 h. The protein levels of TLR4 (**a**), IκBα (**b**), and p65 (**c**) were examined using Western blot assay. (**d**) The nucleus translation of p65 (red) was visualized using immunofluorescence (400×). The nuclei were counterstained with DAPI (blue). The data are expressed as means  ±  SEM (*n* = 6). # *p* < 0.05 vs. LPS (−)/isaridin E (−) group; * *p* < 0.05 vs. LPS (+)/isaridin E (−) group.

As illustrated in Figure 5, LPS stimulation (1 μg/mL) significantly enhanced TLR4 expression in HUVECs compared to the untreated control. Isaridin E intervention dose-dependently attenuated this response, with TLR4 levels reduced by 66.2 ± 4.3% at 24 μm. Concurrently, isaridin E upregulated the expression of IκBα, which is the endogenous inhibitor of NF-κB, in a dose-dependent manner. This increase in IκBα was associated with a corresponding decrease in phosphorylated NF-κB p65 levels, which is indicative of suppressed NF-κB activation. The optimal inhibitory effect on NF-κB phosphorylation was achieved at 24 μm isaridin E. These findings suggest that isaridin E mitigates LPS-induced endothelial inflammation by disrupting the TLR4/NF-κB signaling axis.

To further elucidate the mechanism by which isaridin E suppresses endothelial inflammation, we performed immunofluorescence staining for the NF-κB p65 subunit in HUVECs. NF-κB p65 was labeled with Alexa Fluor 594 (red fluorescence), and nuclei were counterstained with DAPI (blue fluorescence). Confocal microscopy revealed a diffuse cytoplasmic distribution of p65 in control cells, with minimal nuclear localization. LPS stimulation induced a robust nuclear translocation of p65, as evidenced by a marked elevation of red fluorescence in the nucleus. Conversely, this nuclear translocation, which is a hallmark of NF-κB activation, was significantly impeded by isaridin E treatment at 24 μm (Figure 5d). These data demonstrate that isaridin E suppresses NF-κB signaling in LPS-activated endothelial cells by impairing p65 nuclear translocation. This aligns with our earlier observations of reduced inflammatory cytokine production (Figure 2), collectively supporting a critical role for isaridin E in disrupting the TLR4/NF-κB inflammatory cascade.

### 2.6. Isaridin E Downregulated LPS-Induced TLR4 Expression and NF-κB Activation In Vivo

**Figure 6:**
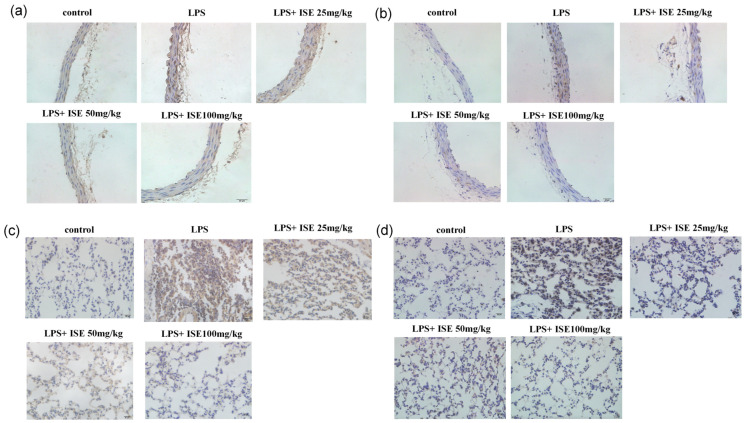
Effects of isaridin E on LPS-induced TLR4 expression and NF-κB activation in vivo. Isaridin E attenuated LPS-induced TLR4 (**a**,**c**) and phosphorylated p65 (**b**,**d**) expression in the aorta and lung tissues detected using immunohistochemistry (400×). Yellowish-brown reaction product referred to TLR4 and p65 positive distribution, *n* = 5.

To further validate the anti-inflammatory mechanism of isaridin E in vivo, we performed an immunohistochemical analysis of TLR4 and phosphorylated p65 (p-p65) in aortic and lung tissues from LPS-challenged mice. Immunohistochemical staining showed that in comparison to the control group, the LPS-treated animals displayed notably darker brown–yellow staining in both aortic sections (Figure 6a) and lung tissues (Figure 6c), suggesting a marked increase in TLR4 expression, which was dose-dependently attenuated by isaridin E treatment.

Similarly, LPS stimulation resulted in robust p-p65 staining in both the intima–media layer of the aorta and lung parenchyma (Figure 6b,d). In contrast, isaridin E treatment at tested doses significantly reduced p-p65 levels in both tissues. These data demonstrate that isaridin E effectively suppresses TLR4 expression and NF-κB activation in vascular and pulmonary tissues, providing in vivo evidence for its therapeutic potential in mitigating LPS-induced inflammation.

## 3. Discussion

In the present study, we demonstrated the robust anti-inflammatory efficacy of isaridin E in both in vitro and in vivo models of LPS-induced endothelial inflammation. Notably, our findings establish isaridin E as a novel natural marine metabolite with significant therapeutic potential for mitigating endothelial inflammation during LPS-induced endotoxemia. Specifically, we show that isaridin E attenuates LPS-driven inflammatory responses in vascular and pulmonary tissues by disrupting the TLR4/NF-κB signaling cascade, a pivotal pathway in the pathogenesis of sepsis and other inflammatory diseases.

Endothelial cells, strategically positioned at the interface of blood and vessel walls, serve as dynamic sensors and effectors in inflammatory responses [9,10]. While not viewed as classical immune cells, upon infection or other injury, they orchestrate immune activation through the secretion of various pro-inflammatory cytokines (e.g., TNF-α and IL-6) and chemokines (e.g., MCP-1). Furthermore, endothelial cells are critical for mediating leukocyte trafficking into tissues via the upregulation of adhesion molecule expression (such as ICAM-1 and VCAM-1) on their surface [11,12,13]. Endothelial dysfunction has been regarded as a central pathophysiological feature in sepsis, atherosclerosis, and other inflammation-driven diseases [1,2,3]. Consequently, targeting endothelial activation represents a promising therapeutic strategy to mitigate systemic inflammation and organ injury. The search for novel therapeutic agents to reduce endothelial-mediated inflammation is an area of intense research.

In the present study, utilizing a widely used LPS-induced inflammation model, we provide compelling evidence that isaridin E, a cyclic hexapeptide compound derived from the marine fungus *Beauveria felina*, potently suppresses LPS-triggered inflammatory responses in HUVECs and in a murine endotoxemia model. The administration of isaridin E markedly decreased the LPS-induced secretion of pro-inflammatory cytokines and adhesion molecule expression, accompanied with a marked reduction in monocyte adhesion to activated endothelial monolayers, which is a critical step in leukocyte extravasation during inflammation. In line with the in vitro findings, the administration of isaridin E to LPS-challenged mice profoundly suppressed the aortic expression of TNF-α and ICAM-1, with a remarkable improvement in vascular architecture. Importantly, the anti-inflammatory effects of isaridin E were achieved without overt cytotoxicity (Figure 1). Together with the previous finding that isaridin E inhibits neutrophil activation [6], the beneficial effects of this compound on endothelial cells collectively position isaridin E as a promising candidate for therapeutic intervention in inflammatory vascular diseases.

Accumulating evidence shows that endothelial hyperpermeability is a key driver of inflammation-driven pathologies. During inflammatory states, endothelial cells respond to stimuli like LPS by secreting cytokines and upregulating adhesion molecules, which increase vascular permeability. This disruption of endothelial barrier function is particularly evident in the lung, where increased permeability contributes to pulmonary edema and inflammatory cell infiltration, which are hallmarks of LPS-induced lung injury [14,15]. Experimental approaches targeting endothelial integrity have proven to be promising therapies during sepsis [16,17]. In a recent study from our group, we observed that isaridin E pretreatment significantly ameliorated pulmonary vascular permeability in a CLP-induced sepsis model. This protective effect was mechanistically linked to a significant reduction in vWF levels and a suppression of platelet–endothelial interactions [8]. To further characterize the therapeutic potential of isaridin E in inflammatory diseases, we evaluated its ability to protect lung tissue from LPS-induced damage. In line with previous findings [8], our results demonstrate that isaridin E significantly attenuates LPS-induced endothelial hyperpermeability in the lung, as quantified by reduced Evans Blue dye extravasation into the lung interstitium. This preservation of endothelial barrier integrity was accompanied by a marked reduction in LPS-stimulated cytokine (TNF-α) and adhesion molecule (ICAM-1) expression within the lung tissue. Moreover, isaridin E treatment effectively suppressed inflammatory cell transmigration and blood cell extravasation into the lung parenchyma, as evidenced by histological analysis. These findings collectively highlight isaridin E’s capacity to safeguard the vascular endothelium against LPS-induced injury, thereby mitigating downstream organ dysfunction.

Emerging evidence underscores the TLR4/NF-κB signaling axis as a central regulator of inflammatory pathologies, including chronic inflammation and sepsis [18]. The stimulation of endothelial cells by LPS activates TLR4, which triggers a downstream cascade that culminates in NF-κB activation [19,20]. NF-κB, which is a crucial transcription factor acting as a switch to regulate early gene expression, is critically implicated in the pathogenesis of inflammation and immune responses [21,22,23]. Numerous studies have shown that the inhibition of the TLR4/NF-κB pathway alleviates the inflammatory response of endothelial cells [24,25,26]. Given the pivotal role of TLR4/NF-κB signaling in endothelial inflammation, we investigated whether isaridin E modulates this pathway. Our findings demonstrated that isaridin E significantly suppressed TLR4 expression and inhibited NF-κB p65 phosphorylation and nuclear translocation in LPS-stimulated HUVECs, as demonstrated using immunoblotting and confocal microscopy. These in vitro findings were recapitulated in vivo, where immunohistochemical analysis revealed that isaridin E treatment reduced TLR4 expression and NF-κB p65 phosphorylation in the aortic and lung tissues from LPS-challenged mice. Collectively, these results suggest that isaridin E attenuates endothelial inflammation, at least in part, by suppressing the TLR4/NF-κB signaling cascade.

**Figure 7:**
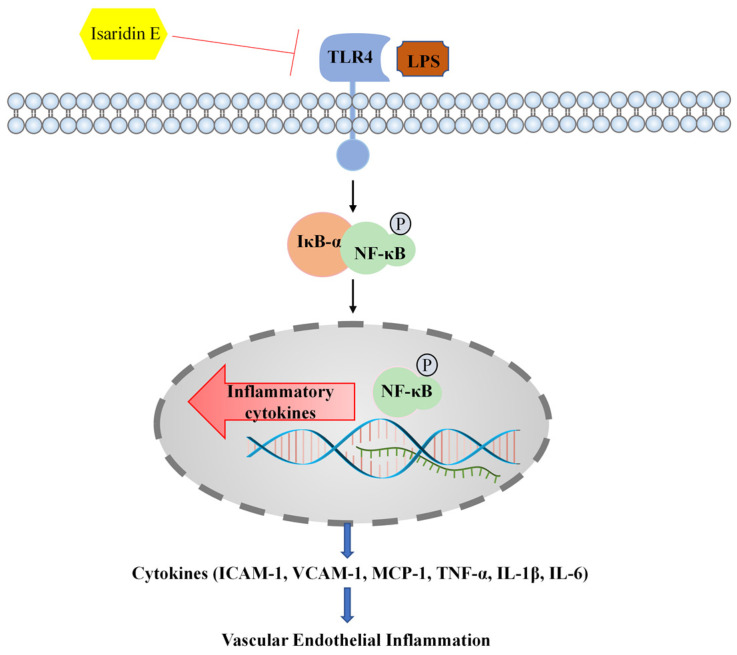
Schematic overview of this study. Isaridin E inhibits the activation of the LPS-induced TLR4/NF-κB signaling pathway, thereby reducing the production of pro-inflammatory cytokines and alleviating vascular endothelial inflammation and tissue injury.

In summary, our research has provided evidence demonstrating that isaridin E exerts potent anti-inflammatory effects against LPS-induced endothelial inflammation in both cellular and preclinical models. Mechanistically, these effects are attributed to the suppression of TLR4/NF-κB signaling, which is a critical pathway driving inflammatory responses in sepsis and other vascular pathologies. Consequently, isaridin E may emerge as a potential therapeutic agent to mitigate inflammatory tissue injury for conditions characterized by endothelial inflammation or TLR4/NF-κB dysregulation. A schematic overview of this study is shown in Figure 7.

## 4. Materials and Methods

### 4.1. Materials

Isaridin E was isolated from the ascidian-derived fungal strain *Amphichorda felina* (syn. *B. felina*) sp. SYSU-MS7908, as described previously by the School of Marine Sciences, Sun Yat-Sen University [7]. Isaridin E was dissolved in dimethyl sulfoxide (DMSO, at a final concentration of less than 0.1%) and stored at 4 °C, before being diluted to different concentrations as needed for in vitro experiments. For intragastric administrations, isaridin E was dissolved in a saline solution containing 10% Tween 80 and 15% propylene glycol to 2.5, 5, and 10 mg/mL, respectively. LPS was purchased from Sigma-Aldrich (Saint Louis, MO, USA).

### 4.2. Animals

Male SPF-grade C57BL/6 mice, aged 8–10 weeks and weighing approximately 20–25 g, were supplied by the Experimental Animal Center of Zhongshan Medical School, Sun Yat-Sen University in Guangzhou, China. Animal experiments were approved by the Animal Care and Use Committee of Sun Yat-Sen University (Approval No: SYSU-IACUC-2021-B0064), in accordance with the relevant regulations on animal welfare and ethics for experimental animals in China.

Mice were fed a standard diet and randomly divided into 5 groups. Vascular inflammation was induced by LPS injection (intraperitoneally; 15 mg/kg), and mice were sacrificed after 12 h. To examine the effects of isaridin E, mice were intragastrically administrated multiple doses of vehicle or isaridin E (25, 50, or 100 mg/kg) for 3 days before LPS injection. Mice injected intraperitoneally with 15 mg/kg saline were used as controls.

### 4.3. Cell Culture and Treatment

The experiments were approved by the medical research ethics committee of Sun Yat-Sen University and were conducted according to the principles expressed in the Declaration of Helsinki. Informed consent was obtained from all subjects. In brief, HUVECs were harvested from the umbilical vein and digested by 0.125% trypsin (Sigma-Aldrich, USA) with 0.01% ethylene glycol-bis (β-aminoethyl ether)-N,N,N’,N’-tetraacetic acid (EGTA). After that, the cells were cultured in M199 culture medium (Sigma-Aldrich, USA) containing 20% fetal calf serum, 100 U/mL penicillin, 100 U/mL streptomycin, 25 U/mL heparin, 2 mmol/L L-glutamine, and 5 ng/mL p-ECGF at 37 °C under a 5% CO_2_-humidified atmosphere. Cells between passages 4 and 8 were used in this study.

### 4.4. Monocyte Adhesion Assay

THP-1 cells (Procell, Wuhan, China) were labeled with 3 μm Calcein-AM (Beyotime Biotechnology, Haimen, China) for 30 min at 37 °C in 5% CO_2_. HUVECs pretreated with isaridin E (6 μm, 12 μm, 24 μm, 48 μm, and 96 μm) for 2 h were plated in 35 mm culture dishes at a density of 2 × 10^5^ cells/mL. After incubation with LPS (1 μg/mL) for 18 h, the cells were washed twice; then, the Calcein-AM-labeled monocytes were added into each culture dish for 1 h at 37 °C, under 5% CO_2_. To remove non-adherent cells, the dishes were gently washed with pre-warmed RPMI-1640. This was repeated twice and then 1 mL RPMI-1640 medium was added into each dish and the images were captured using Olympus Fluoview 500 laser confocal scanning microscopy with an excitation wavelength of 485 nm and emission at 530 nm. At least 6 fields randomly selected in each dish were observed.

### 4.5. Quantitative Real-Time PCR

Quantitative RT-PCR was performed following the manufacturer’s instructions. Briefly, total RNA was extracted using Trizol reagent (Invitrogen, Waltham, MA, USA); then, the RNA (500 ng) was used for reverse transcription using the PrimeScript RT reagent Kit Perfect Real-Time kit (Takara Bio Inc., Shiga, Japan). The cDNA was used for quantitative real-time PCR analysis using SYBR Green reagents (Takara Bio Inc., Japan). The 2ΔΔCt method was applied for the relative quantification of the target mRNA. The primers used for human IL-6 are forward AGACAGCCACTCACCTCTTCAG and reverse TTCTGCCAGTGCCTCTTTGCTG. The primers used for human TNF are forward CTCTTCTGCCTGCTGCACTTTG and reverse ATGGGCTACAGGCTTGTCACTC. The primers used for human VCAM-1 are forward GATTCTGTGCCCACAGTAAGGC and reverse TGGTCACAGAGCCACCTTCTTG. The primers used for human ICAM-1 are forward GTATGAACTGAGCAATGTGCAAG and reverse GTTCCACCCGTTCTGGAGTC. The primers used for human MCP-1 are forward ACGGGATCGTGGTTTCCAAC and reverse TGGCTTCTTACGCAGGAAGTT. The primers used for human IL-1β are forward ATGATGGCTTATTACAGTGGCAA and reverse GTCGGAGATTCGTAGCTGGA. All primers were synthesized by Guangzhou Huiyuan Biotechnology Co., Ltd. (Guangzhou, China).

### 4.6. Western Blot

Western blot was performed according to the protocol described previously [7,8]. HUVECs were rinsed with ice-cold PBS and lysed with lysis buffer containing a protease inhibitor cocktail (Merck, Darmstadt, Germany). The protein concentration was determined with a BCA kit. Protein was separated using SDS-PAGE and transformed into PVDF membranes (Millipore, Burlington, MA, USA). After blocking in 5% skimmed milk for 1 h at room temperature, the membranes were incubated with primary antibodies against TNF-α (Servicebio, Wuhan, China), ICAM-1 (Servicebio, China), TLR-4 (Proteintech Group, Rosemont, IL, USA), IκBα (Cell Signaling Technology, USA), and p-p65 (Cell Signaling Technology, USA) at 4 °C overnight. Incubation with monoclonal mouse β-actin (Proteintech Group, USA) antibody was performed as the loading sample control. After incubation with the secondary antibody conjugated to horseradish peroxidase (Cell Signaling Technology, Danvers, MA, USA) for 1 h at room temperature, bands were detected using the Pierce ECL Western blotting substrate (Thermo Scientific, Waltham, MA, USA) and were quantified using a computer-aided one-dimensional gel analysis system.

### 4.7. Evans Blue Staining of the Lungs

A 5% working solution of Evans blue was prepared by dissolving it in sterile physiological saline. Upon injection into mice, their bodies exhibited immediate blue discoloration. After a 2 h circulation period, the mice were anesthetized and placed in a supine position on a mouse board. The abdominal and thoracic cavities were surgically opened to expose the heart and lungs. A small incision was made in the left atrium, followed by the injection of chilled PBS solution containing 0.1% sodium heparin into the right ventricle for perfusion. Perfusion was discontinued upon the absence of red blood spots in the lungs. The lungs, stained with Evans blue, were harvested, photographed, and homogenized in centrifuge tubes containing 1 mL of acetamide per 30 mg of lung tissue. The tubes were subsequently incubated in a dark 37 °C chamber for 24 h. Following incubation, the tubes were centrifuged at 14,000 rpm and room temperature for 30 min, and the resulting supernatant was transferred to fresh 1.5 mL EP tubes for future use. The absorbance of the supernatant was quantified at 620 nm using a UV spectrophotometer.

### 4.8. Immunohistochemistry

Mice were anesthetized with 2% pentobarbital sodium and were perfused intracardiacly with Kreb’s solution containing 137 mmol/L NaCl, 5.4 mmol/L KCl, 2.0 mmol/L CaCl_2_, 1.1 mmol/L MgCl_2_, 0.4 mmol/L NaH_2_PO_4_, 5.6 mmol/L Glucose, 11.9 mmol/L NaHCO_3_, and 10 U/mL heparin, followed by 4 °C fixative solution containing 4% freshly depolymerized paraformaldehyde in 0.1 mol/L phosphate buffer for 10–15 min. After the fat and connective tissue was cleaned, the thoracic aorta was embedded and frozen using an optimal cutting temperature compound (OCT, Sakura, Japan). Immunohistochemistry was performed using the streptavidin–biotin–peroxidase complex system, according to the manufacturer’s instructions (SABC Peroxidase Kit, Absin Bioscience, Shanghai, China). Cryostat sections (8 μm) were pretreated with the solution of 3% hydrogenperoxide and methanol at a ratio of 1:50 for 30 min at room temperature, and were then blocked with 5% bovine serum albumin in PBS for 30 min. Then, the sections were incubated with ICAM-1 (1:200 dilution; Santa Cruz Biotechnology, USA) or TNF-α (1:200 dilution; Santa Cruz Biotechnology, USA) polyclonal antibody at 4 °C overnight, and were then treated with a biotinylated secondary anti-rabbit antibody for 30 min at room temperature. After that, the sections were incubated with the streptavidin–biotin–peroxidase complex for 30 min at room temperature. Staining was performed with DAB chromogen. Slides were counterstained with hematoxylin.

### 4.9. Nuclear Translocation of NF-κB

The cells underwent 2–3 washes with PBS before being fixed with 1 mL of 4% paraformaldehyde for 1 h. Following three additional washes with PBS, 1 mL of 1% Triton X-100 was added to permeabilize the cells for 1 h. Subsequently, the cells were incubated with 100 μL of immunostaining blocking solution at 37 °C for 1 h. After removing the excess blocking solution, the cells were incubated with NF-κB p65 antibody (diluted 1:100, Santa Cruz Biotechnology, Dallas, TX, USA) overnight in a humid chamber at 4 °C. After another round of PBS washes, an immunofluorescence secondary antibody solution was added, and the cells were incubated in a dark, humid chamber at 37 °C for 1 h. The cells were then treated with a fluorescence quenching solution containing DAPI and stored in the dark. Images were visualized and captured using a laser scanning confocal microscope.

### 4.10. Statistical Analyses

Blinded data analysis was performed using GraphPad Prism 8.0 (GraphPad software, 20 La Jolla, CA, USA, RRID:SCR_002798). All data were presented as mean ± SEM, and the n value represents the number of independent experiments on different batches of cells or different mice. Statistical analysis was determined using an unpaired two-tailed Student t test or ANOVA followed by a Bonferroni multiple comparison test with a 95% confidence interval. Values of *p* < 0.05 were considered significant.

## Data Availability

All data included in this study are available upon request from the corresponding author.

## References

[1] Barbu E., Popescu M.R., Popescu A.C., Balanescu S.M. (2022). Inflammation as A Precursor of Atherothrombosis, Diabetes and Early Vascular Aging. Int. J. Mol. Sci..

[2] Krüger-Genge A., Blocki A., Franke R.P., Jung F. (2019). Vascular Endothelial Cell Biology: An Update. Int. J. Mol. Sci..

[3] Hellenthal K.E.M., Brabenec L., Wagner N.M. (2022). Regulation and Dysregulation of Endothelial Permeability during Systemic Inflammation. Cells.

[4] Assar M.E., Angulo J., Rodriguez-Manas L. (2016). Diabetes and ageing-induced vascular inflammation. J. Physiol..

[5] Langenfeld A., Blond A., Gueye S., Herson P., Nay B., Dupont J., Prado S. (2011). Insecticidal Cyclodepsipeptides from Beauveria felina. J. Nat. Prod..

[6] Chung Y.M., El-Shazly M., Chuang D.W., Hwang T.L., Asai T., Oshima Y., Ashour M.L., Wu Y.C., Chang F.R. (2013). Suberoylanilide hydroxamic acid, a histone deacetylase inhibitor, induces the production of anti-inflammatory cyclodepsipeptides from Beauveria felina. J. Nat. Prod..

[7] Pan N., Li Z.C., Li Z.H., Chen S.H., Jiang M.H., Yang H.Y., Liu Y.S., Hu R., Zeng Y.W., Dai L.H. (2021). Antiplatelet and Antithrombotic Effects of Isaridin E Isolated from the Marine-Derived Fungus via Downregulating the PI3K/Akt Signaling Pathway. Mar. Drugs.

[8] Liu Y.S., Chen W.L., Zeng Y.W., Li Z.H., Zheng H.L., Pan N., Zhao L.Y., Wang S., Chen S.H., Jiang M.H. (2024). Isaridin E Protects against Sepsis by Inhibiting Von Willebrand Factor-Induced Endothelial Hyperpermeability and Platelet-Endothelium Interaction. Mar. Drugs.

[9] Wang L., Cheng C.K., Yi M., Lui K.O., Huang Y. (2022). Targeting endothelial dysfunction and inflammation. J. Mol. Cell. Cardiol..

[10] Konukoglu D., Uzun H. (2017). Endothelial Dysfunction and Hypertension. Adv. Exp. Med. Biol..

[11] Mittal M., Siddiqui M.R., Tran K., Reddy S.P., Malik A.B. (2014). Reactive oxygen species in inflammation and tissue injury. Antioxid. Redox Signal..

[12] Zhang Y., Igwe O.J. (2018). Lipopolysaccharide (LPS)-mediated priming of toll-like receptor 4 enhances oxidant-induced prostaglandin E2 biosynthesis in primary murine macrophages. Int. Immunopharmacol..

[13] Ishii M., Nakahara T., Araho D., Murakami J., Nishimura M. (2017). Glycolipids from spinach suppress LPS induced vascular inflammation through eNOS and NK-kappaB signaling. Biomed. Pharmacother..

[14] Klein O.R., Ktena Y.P., Pierce E., Fu H.H., Haile A., Liu C., Cooke K.R. (2023). Defibrotide modulates pulmonary endothelial cell activation and protects against lung inflammation in pre-clinical models of LPS-induced lung injury and idiopathic pneumonia syndrome. Front. Immunol..

[15] Wang L., Cao Y., Gorshkov B., Zhou Y., Yang Q., Xu J., Ma Q., Zhang X., Wang J., Mao X. (2019). Ablation of endothelial Pfkfb3 protects mice from acute lung injury in LPS-induced endotoxemia. Pharmacol. Res..

[16] Joffre J., Hellman J., Ince C., Ait-Oufella H. (2020). Endothelial Responses in Sepsis. Am. J. Respir. Crit. Care Med..

[17] Liu Y., Mu S., Li X., Liang Y., Wang L., Ma X. (2019). Unfractionated Heparin Alleviates Sepsis-Induced Acute Lung Injury by Protecting Tight Junctions. J. Surg. Res..

[18] Zuliani-Alvarez L., Marzeda A.M., Deligne C., Schwenzer A., McCann F.E., Marsden B.D., Piccinini A.M., Midwood K.S. (2017). Mapping tenascin-C interaction with toll-like receptor 4 reveals a new subset of endogenous inflammatory triggers. Nat. Commun..

[19] Shirai T., Hilhorst M., Harrison D.G., Goronzy J.J., Weyand C.M. (2015). Macrophages in vascular inflammation—From atherosclerosis to vasculitis. Autoimmunity.

[20] Ali L., Schnitzler J.G., Kroon J. (2018). Metabolism: The road to inflammation and atherosclerosis. Curr. Opin. Lipidol..

[21] Zusso M., Lunardi V., Franceschini D., Pagetta A., Lo R., Stifani S., Frigo A.C., Giusti P., Moro S. (2019). Ciprofloxacin and levofloxacin attenuate microglia inflammatory response via TLR4/NF-kB pathway. J. Neuroinflamm..

[22] Kempe S., Kestler H., Lasar A., Wirth T. (2005). NF-kappaB controls the global pro-inflammatory response in endothelial cells: Evidence for the regulation of a pro-atherogenic program. Nucleic Acids Res..

[23] Lei J., Xiang P., Zeng S., Chen L., Zhang L., Yuan Z., Zhang J., Wang T., Yu R., Zhang W. (2022). Tetramethylpyrazine Alleviates Endothelial Glycocalyx Degradation and Promotes Glycocalyx Restoration via TLR4/NF-κB/HPSE1 Signaling Pathway During Inflammation. Front. Pharmacol..

[24] Wu Y., Wang Y., Gong S., Tang J., Zhang J., Li F., Yu B., Zhang Y., Kou J. (2020). Ruscogenin alleviates LPS-induced pulmonary endothelial cell apoptosis by suppressing TLR4 signaling. Biomed. Pharmacother..

[25] Jin Y., Nguyen T.L.L., Myung C.S., Heo K.S. (2022). Ginsenoside Rh1 protects human endothelial cells against lipopolysaccharide-induced inflammatory injury through inhibiting TLR2/4-mediated STAT3, NF-κB, and ER stress signaling pathways. Life Sci..

[26] Huang L., Li Y., Cheng Z., Lv Z., Luo S., Xia Y. (2023). PCSK9 Promotes Endothelial Dysfunction During Sepsis Via the TLR4/MyD88/NF-κB and NLRP3 Pathways. Inflammation.

